# LESM-YOLO: An Improved Aircraft Ducts Defect Detection Model

**DOI:** 10.3390/s24134331

**Published:** 2024-07-03

**Authors:** Runyuan Wen, Yong Yao, Zijian Li, Qiyang Liu, Yijing Wang, Yizhuo Chen

**Affiliations:** 1 School of Computer Science and Technology, Xidian University, Xi’an 710126, China; wenrunyuan@stu.xidian.edu.cn (R.W.); zjian.li@stu.xidian.edu.cn (Z.L.); 22031212452@stu.xidian.edu.cn (Q.L.); 21031211499@stu.xidian.edu.cn (Y.W.); 2 Guangzhou Institute of Technology, Xidian University, Guangzhou 510530, China; yizhuo@stu.xidian.edu.cn

**Keywords:** aircraft ducts, YOLOv8, technical diagnostics, defect detection

## Abstract

Aircraft ducts play an indispensable role in various systems of an aircraft. The regular inspection and maintenance of aircraft ducts are of great significance for preventing potential failures and ensuring the normal operation of the aircraft. Traditional manual inspection methods are costly and inefficient, especially under low-light conditions. To address these issues, we propose a new defect detection model called LESM-YOLO. In this study, we integrate a lighting enhancement module to improve the accuracy and recognition of the model under low-light conditions. Additionally, to reduce the model’s parameter count, we employ space-to-depth convolution, making the model more lightweight and suitable for deployment on edge detection devices. Furthermore, we introduce Mixed Local Channel Attention (MLCA), which balances complexity and accuracy by combining local channel and spatial attention mechanisms, enhancing the overall performance of the model and improving the accuracy and robustness of defect detection. Finally, we compare the proposed model with other existing models to validate the effectiveness of LESM-YOLO. The test results show that our proposed model achieves an mAP of 96.3%, a 5.4% improvement over the original model, while maintaining a detection speed of 138.7, meeting real-time monitoring requirements. The model proposed in this paper provides valuable technical support for the detection of dark defects in aircraft ducts.

## 1. Introduction

Aircraft ducts are an important component of normal aircraft operation, and the timely detection and maintenance of aircraft ducts are crucial for safe aircraft operation [1]. Defects in aircraft ducts have a significant impact on their performance, and the toughness, strength, and corrosion resistance of aircraft ducts are important factors to ensure their safety. If cracks, deformations, or corrosion are present in the conduit, these conditions will weaken its toughness and strength, making it more prone to fracture and damage during use. The traditional defect detection of aircraft ducts is mostly based on manual handheld endoscopic inspection. Aircraft ducts are small, and the detection time is insufficient, which leads to workers needing to perform fault detection in a narrow environment, which is time-consuming and labor-intensive, and highly dependent on the workers’ prior experience. The detection process has high labor intensity, a low detection efficiency, and a high probability of detection errors.

Currently, defect detection in aircraft ducts faces the following major challenges. Firstly, high-precision detection is crucial. The safety of aircraft ducts is of the utmost importance, so the accuracy requirements for defect detection systems are very high. The system must be able to accurately detect any potential defects or anomalies to avoid potential safety risks [2]. Additionally, the detection process is complicated by diverse environments and backgrounds. Different materials are used for aircraft ducts in different aircrafts, some of which have reflective properties. If LED light sources are solely incorporated into the collection equipment, scratch defects in the reflective aircraft ducts are likely to be overlooked. Furthermore, during operation, stains are often present, which can easily be confused with defects in the ducts. Another issue is the low quality of the existing data. Since aircraft ducts are small aircraft components, the collected data often suffer from insufficient lighting. Direct detection in low-light environments fails to meet the high-precision defect detection requirements, and the data contain significant noise. Therefore, it is necessary to develop an automatic, efficient, and economical inspection framework for aircraft ducts to reduce reliance on manual detection, decrease the difficulty of the task, detect defects early, prevent catastrophic failures, and minimize maintenance downtime [3].

To address the aforementioned issues and improve the detection of small defects in low-light environments, as well as to enable the lightweight deployment of the model on edge computing devices, we propose a novel object detection framework, Light Enhancement defect detection based on Space-to-depth convolution and Mixed-channel attention (LESM)-YOLO. In the light enhancement module, we utilized shared weights and a layer-by-layer connection approach to optimize the illumination components, thereby improving image exposure. The self-calibration module ensures the stable convergence of results at each stage. Additionally, we optimized the dataset to address the issue of low-quality defect images captured in low-light conditions. We replaced the conventional convolution module in YOLOv8 with the space-to-depth convolution module, which transforms spatial information into depth information. This effectively reduces information loss and preserves more detailed features, thereby enhancing the accuracy of feature extraction. Given the prevalence of small target defects in aircraft duct inspection, substituting traditional stride convolution and pooling layers with this module significantly improves defect detection accuracy while reducing the number of parameters, meeting the requirements for high-precision detection. Finally, we employed a hybrid channel attention mechanism to address the issue of neglected spatial feature information in the existing CBAM [4] attention mechanism. This approach integrates channel and spatial information with only a slight increase in parameter count, effectively dealing with the complex environments and backgrounds in aircraft duct defect detection. By using this attention mechanism, irrelevant modules are suppressed, significantly enhancing detection performance. Comprehensive experiments demonstrate that LESM-YOLO outperforms all comparative models in terms of mAP metrics.

Our contributions can be summarized as follows:

1. By analyzing the challenges in detecting defects in aircraft ducts under low-light conditions, we integrated a light enhancement module. This integration addresses the issue of low-quality defect images captured in low-light environments from a model perspective.
2. By examining the characteristics of existing aircraft duct defects, we replaced the standard convolution modules with SPDConv modules. This effectively reduces information loss and preserves more detailed defect features.
3. To address the complex environments and backgrounds present in aircraft duct defect detection, we incorporated an MLCA into the neck module, significantly enhancing the model’s detection performance.

## 2. Related Work

In recent years, significant progress has been made in the field of object detection due to the emergence of convolutional neural networks and attention mechanisms. Object detection algorithms can be divided into two categories: single-stage object detection algorithms and two-stage object detection algorithms. The common single-level algorithms are SSD [5] and YOLO [6]. The single-stage algorithm treats defect localization and classification as a regression problem, achieving end-to-end detection and a fast detection speed. However, due to class imbalances and other reasons, the accuracy of single-stage algorithms is slightly lower than that of two-stage algorithms. The common two-stage object detection algorithms are Fast R-CNN [7] and Faster R-CNN [8]. These algorithms first generate a region of interest (ROI), and then classify and locate it in the second stage, resulting in high accuracy but a relatively slow detection speed.

The attention mechanisms in the field of computer science mainly include spatial attention [4,9,10], channel attention [11], dynamic convolution filters, etc. Among them, spatial attention is mainly used to capture image spatial information, such as in object detection, image segmentation, and other tasks [12]. The spatial attention mechanism can help the model focus more on the specific channel information of the image [13]. Channel attention is more commonly applied to tasks that require the processing of image channel information, such as image stylization [14], image super-resolution [15,16], etc. Su et al. [17] utilized channel attention mechanisms to enhance the expressive ability of target features, applying them in the field of object tracking. Dynamic convolution filters are more commonly used in tasks such as multitasking learning [18] and model compression, to help models focus more on the key information of the task. However, spatial attention does not pay enough attention to channel information and cannot achieve optimal results for images with insufficient lighting and low resolution. Channel attention also has the problem of insufficient attention to spatial dimensions. Dynamic convolutional filters have high computational costs and are not suitable for learning single tasks. The above attention mechanisms have certain limitations, which make it difficult to apply them well to the detection of defects in aircraft ducts.

Due to the relatively narrow research field on aircraft duct defects and the lack of relevant research results, and considering that aircraft ducts are steel objects with small detection targets, we will conduct more in-depth research on steel object defect detection and small object detection. In terms of model improvements, Wang et al. [19] improved the YOLOv8s model by adding a small object detection layer to address the issue of small object detection loss in the YOLOv8 model, focusing on the problem of small gear defects in automobiles. Li et al. [20] proposed a novel lightweight convolutional technique called GSConv, which could be applied to lightweight models while maintaining accuracy. The algorithm based on deep learning has been widely applied to establish a reliable steel surface defect detection system, which has guiding significance for aircraft duct defect detection. The performance of deep learning models heavily relies on rich annotated data. However, the volume of labeled images in industrial datasets is often limited. To address this issue, Wang et al. [21] proposed the first few-sample defect detection framework. By using data related to the target task for pre-training the model, the proposed framework can generate well-trained networks with a small number of labeled images, and a noise regularization strategy was designed to significantly improve the robustness of the training model. Zhang et al. [22] proposed a dense non-anchored rotating-object detector (DARDet) for detecting rotating objects in aerial images to address the issue of the sensitivity of rotation detection to anchoring parameters and potential performance degradation due to boundary discontinuities. They also introduced a loss of PIoU to achieve accurate and stable regression. This method achieves a state-of-the-art performance while maintaining high efficiency on three commonly used aerial object datasets, namely DOTA, HRSC2016, and UCAS-AOD. Wang et al. [23] designed an efficient anchor-free rotating-object detector based on PP-YOLOE. Many useful techniques have been introduced to PP-YOLOE-R to improve the detection accuracy with few additional parameters and computational costs. The results showed that PP-YOLOE-Rl and PP-YOLOE-R-x achieved 78.14 and 78.28 mAP, respectively, on the DOTA 1.0 dataset, which were almost superior to all other rotating object detectors. Although the above research has made contributions to steel surface defect detection tasks and lightweight models, there is no algorithm with high robustness and accuracy in aircraft duct defect detection. Therefore, we have proposed an innovative defect detection framework based on the anchor-free model, which uses the mixed-channel attention mechanism to enhance the problem of spatial feature information extraction and greatly improve the model accuracy. In addition, we added the light enhancement module to ensure the robustness of the model in a low-illumination detection environment, and used the space-to-depth revolution module to reduce the amount of computation while reducing the loss of information, so that our framework can be well deployed in edge computing equipment to achieve an outstanding performance and ensure that the framework is lightweight, accurate, and robust.

## 3. Proposed Method

**Figure 1:**
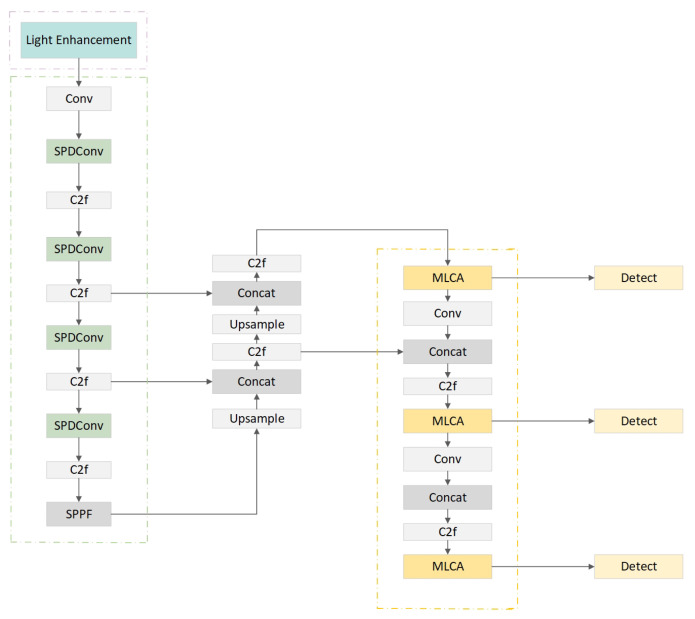
Architecture of LESM-YOLO. We added the light enhancement module before the convolution operation, changed the original Conv convolution in the backbone to SPDConv convolution, and finally added a lightweight MLCA attention mechanism in the neck to enhance the model’s ability to extract spatial feature information.

The overall structure of LESM-YOLO is shown in Figure 1, which comprises three key modules: a low-light enhancement module, an SPDConv-based Bbckbone module, and an MLCA-based neck module. The original YOLOv8 model lacks the ability to identify defects in data collected under low-light conditions and tends to overlook certain details. To overcome these limitations, this study introduces the SPDConv-based backbone module [24,25] and MLCA-based [26] neck module. While reducing the number of parameters to make the model more lightweight, it also takes into account spatial feature information and performs low-light enhancement processing. This enables the efficient and precise detection of small target defects in aircraft ducts, even with unchanged inputs. In this chapter, we will take a closer look at what each module does and how it works.

### 3.1. Low-Light Enhancement Module

The collection of data from aircraft ducts mostly takes place under low-light conditions. Due to the influence of insufficient lighting on the collected data, the quality and clarity of the images may decrease, making it difficult to distinguish defects in the dataset. At the same time, the information captured by the camera will be limited, resulting in a lack of rich details and information in the dataset, which may limit the performance and generalization ability of machine learning models in dark conditions. If low-light datasets are directly annotated, this will lead to errors or inconsistencies being flagged, affecting the quality and reliability of the training data.

**Figure 2:**
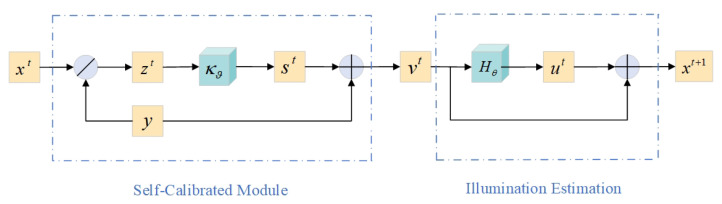
Low-light enhancement module framework.

To overcome the above problems, we reconstructed the YOLOv8 network and introduced a low-light enhancement module [27] into the model’s backbone to optimize image quality. During the training phase, this module includes a self-calibration module and an illumination estimation module, which facilitates weight sharing in illumination learning, causing the results of each stage to converge and thereby improving the exposure stability and significantly reducing computational noise. The structure of the low-light enhancement model is shown in Figure 2.

In the initial stage, the images captured under raw illumination are first processed through the illumination estimation module. In the subsequent t−1 stages, the images from the previous stage are first processed through the self-calibration module before undergoing computation in the illumination estimation module.

In the self-calibrated stage, since the input of each stage comes from the output of the previous stage, the core idea is to combine the input of each stage with weak light observations for calibration to achieve convergence. In the self-calibrated stage, as shown in Figure 2, $x^{t}$ represents the illumination condition at stage *t*, *y* is the low-lightobservation, and $z^{t}$ is the desired clear image at stage *t*. $K_{\theta }$ denotes the shared weights and parameters, and $v^{t}$ is the transformed input after passing through the self-calibration module at stage *t*.

Here, *F* represents the illumination estimation module, which includes the following equations:

(1) $$F\left(x^{t}\right)=\left\{\begin{array}{c} u^{t}=H_{\theta }\left(x^{t}\right)\\ x^{t+1}=x^{t}+u^{t} \end{array}\right.$$

where $H_{\theta }$ represents shared weights, which means that the same architecture *H* and parameters are used at each stage θ. The illumination $x^{t+1}$ in stage t+1 is composed of the residual light $u^{t}$ and illumination $x^{t}$ from the previous stage *t*. The structure also incorporates residual representation to ensure the stability of exposure. In the self-calibrated stage, since the input of each stage comes from the output of the previous stage, the core idea is to combine the input of each stage with weak light observations for calibration to achieve convergence.

The low-light enhancement module uses specific loss functions to maintain the color fidelity, contrast, and detail of the enhanced images. These loss functions ensure that the enhanced images have good visual quality. The loss function is shown in Equation (2):

(2) $$L_{total}=\alpha L_{f}+\beta L_{s}$$

where $L_{f}$ and $L_{s}$ represent fidelity and smoothness losses, respectively. We adopt fidelity loss [27], represented as follows:

(3) $$L_{f}=\sum_{t=1}^{T}\left|\right|x^{t}-\left(y+s^{t-1}\right){\left|\right|}^{2}$$

where $y+s^{t-1}$ is used to constrain the illumination $x^{t}$. At the same time, smoothness loss is a broad consensus in this task [28,29]. The formula is as follows:

(4) $$L_{s}=\sum_{i=1}^{N}\sum_{j\in N(i)}w_{i,j}\left|x_{i}^{t}-x_{j}^{t}\right|$$

### 3.2. SPDConv-Based Backbone

Convolutional neural networks (CNNs) have achieved significant results in various fields, such as image classification, object detection, image segmentation, and medical image analysis. However, in difficult tasks with a low image resolution or small objects, such as when identifying small targets such as cracks, scratches, and defects in aerial ducts, there are shortcomings, such as information granularity loss and a large parameter quantity. Therefore, we introduced space-to-depth convolution (SPDConv) [24,25] to optimize the original YOLOv8 network model architecture so that the model can reduce the number of parameters while maintaining the accuracy and speed of its defect detection.

In the backbone section of YOLOv8, the original convolution operations primarily focus on local features, with the output feature map size determined by the input size, kernel size, stride, and padding. This approach may overlook global contextual information, which is crucial for the classification of defects in aircraft ducts. SPDConv is mainly used for reordering feature maps, allowing spatial information to be transformed into depth information while preserving the original details. This operation is typically undertaken to enhance the expressive capacity of feature maps, making it easier for the model to capture complex spatial relationships. It is not a process of weighted summation, but rather a reorganization process. By reorganizing the data structure of the feature map, its shape is altered to better integrate features from different levels.

**Figure 3:**
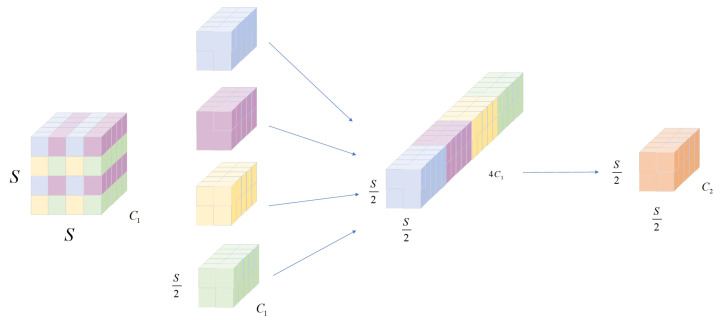
Principle diagram of space to depth convolution.

LESM-YOLO modified the original YOLOv8 model by replacing the original convolution module with a new convolution module consisting of space-to-depth and one-strided convolution layers, based on the original backbone, to eliminate the convolution and pooling layers in each layer. This convolution operation divides the input tensor into blocks according to their spatial dimensions and rearranges them, increasing their depth direction while reducing their spatial direction. The space to depth convolution structure is shown in Figure 3. The specific implementation method is as follows: first, the input tensor is divided according to the specified spatial block size, and each block is rearranged into deeper tensors. This process will reduce the spatial dimensions of the input tensor and increase the depth dimensions. For example, if the size of the input tensor is (*B*, *S*, *S*, $C_{1}$), where *B* represents the batch size, *S* represents the height and width, and $C_{1}$ represents the number of channels, then the tensor size after spatial partitioning and rearrangement may become (*B*, $\frac{S}{k}$, $\frac{S}{k}$, $C_{1}$×$k^{2}$), where *k* is the size of the spatial partitioning. Finally, a convolutional layer with a stride of 1 is used, the number of channels is set to $C_{2}$, and a stride of 1 convolution is used to preserve all feature information. Finally, the image output is (*B*, $\frac{S}{k}$, $\frac{S}{k}$, $C_{2}$). Space-to-depth convolution can effectively preserve feature information with fewer parameters and lower computational costs than the original convolution. By utilizing the sparsity of spatial information and reorganizing in the depth direction to reduce computational complexity, it can accurately detect small defects and can be well applied in aircraft duct defect detection problems.

### 3.3. MLCA-Based Neck

Currently, the enhancement of object detection models mainly focuses on loss function optimization, network structure optimization, and data augmentation. The attention mechanism is a key component of network structure optimization, as it helps the model better focus on important parts of the image and suppress irrelevant elements, thereby improving detection accuracy and efficiency. However, most channel attention mechanisms only include feature information and ignore spatial feature information, which can lead to poor model representation or a poor object detection performance. Some attention mechanisms do incorporate spatial information, but their computational and parameter requirements are too high, which often results in reduced accuracy due to channel dimension reductions during fusion. To achieve a balance between accuracy and complexity in aircraft duct defect detection and address the issues present in the aforementioned attention mechanisms, this paper introduces a neck module based on mixed local channel attention (MLCA-based neck).

This module consists of two parts: local channel attention and spatial attention. Local channel attention enhances the discriminative power of feature maps by emphasizing important channels and suppressing irrelevant ones. It calculates channel attention weights through global average pooling (GAP) and a series of fully connected layers. Spatial attention aims to highlight important regions within each feature map by aggregating spatial information through convolutional layers and generating a spatial attention map.

The MLCA-based neck module primarily consists of the following steps: First, feature maps are extracted from the backbone network, denoted as $F\in R^{C\times H\times W}$, where *C* is the number of channels, and *H* and *W* are the height and width of the feature maps, respectively. In the local channel attention mechanism part, global average pooling is applied to the feature maps to obtain channel descriptors, as shown in Equation (5). These descriptors are passed through a small feed-forward network (usually consisting of one or two fully connected layers) to compute the channel attention weights, as shown in Equation (6).

(5) $$f_{c}=\frac{1}{H\times W}\sum_{i=1}^{H}\sum_{j=1}^{W}F_{cij}$$

(6) $$M_{c}=\sigma \left(W_{1}\delta \left(W_{0}f_{c}\right)\right)$$

Here, σ is the sigmoid activation function, δ is the ReLU activation function, and $W_{0}$ and $W_{1}$ are learnable weight matrices.

As shown in Equation (7), the spatial attention mechanism applies convolutional layers (usually with a 7 × 7 kernel) to the feature maps to capture spatial dependencies and generate spatial attention maps.

(7) $$M_{s}=\sigma \left(Conv\left(F\right)\right)$$

where Conv represents the convolution operation and σ is the sigmoid activation function.

Finally, the original feature maps are combined with the computed channel and spatial attention maps to enhance the features, as expressed in Equation (8). This design allows for the simultaneous consideration of both channel and spatial information, enhancing relevant features while suppressing irrelevant ones.

(8) $$F^{\prime }=F\times M_{c}\times M_{s}$$

## 4. Experiments and Analysis

### 4.1. Experimental Environment

To verify the effectiveness of our proposed method, we used the Ubuntu operating system, Pytorch 2.1.0 as the deep learning framework, and YOLOv8s as the baseline network model. The specific configuration of the experimental environment is shown in Table 1.

During the training phase, we adopted hyperparameter settings as shown in Table 2.

### 4.2. Dataset and Evaluation Metrics

**Figure 4:**
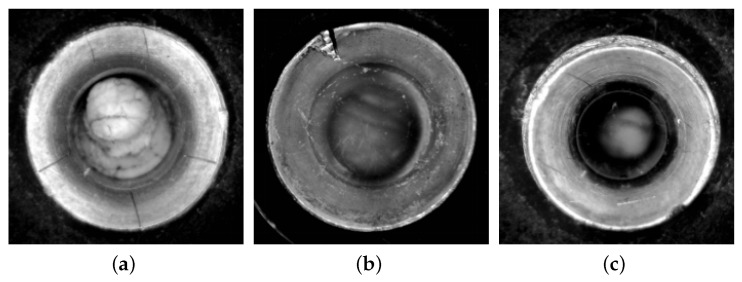
Different defect collection samples. The main defect in (**a**) is cracks, the main defect in (**b**) is defects, and the main defect in (**c**) is scratches. Each image may also contain multiple types of defect.

In this study, a specially designed instrument was used to collect data on internal defects in aircraft ducts. The images are grayscale with a resolution of 1100 × 1100, and a total of 1800 images were collected. For aircraft ducts of varying diameters, we employed different diameter-specific acquisition devices to complete the data collection. In the dataset of 1800 images, the training set contains 1250 images, the validation set contains 270 images, and the test set contains 280 images. Among these, there are 1746 cracks, 2153 scratches, and 2483 defects. Each image reflects different defect issues in the actual use of aircraft ducts. To ensure the accuracy of detecting defect locations, the roLabelImg image annotation software was used to manually annotate the defect areas. We classify aircraft duct defects into three categories: scratch, defect, and crack. The annotation file is saved in XML format and then converted to TXT format to adapt to the YOLOv8 algorithm. Figure 4 shows partial images of the dataset.

**Figure 5:**
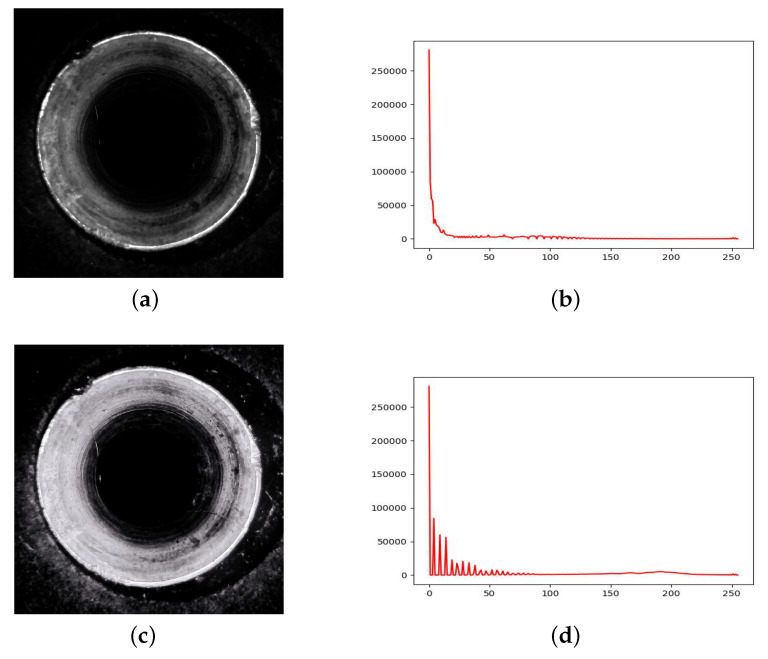
The influence of the lighting enhancement module on the grayscale distribution histogram of images. (**a**) Original image. (**b**) Original grayscale distribution. (**c**) Image after lighting enhancement. (**d**) Grayscale distribution after lighting enhancement.

In addition, we used a grayscale distribution histogram to measure the brightness of the image. It can be seen that the introduction of a lighting enhancement module has an impact on the grayscale distribution histogram of the image, as shown in Figure 5.

In order to objectively evaluate the performance of defect detection in aircraft ducts, this study used Precision (*P*), Recall (*R*), mean Average Precision (mAP)and Frames Per Second (FPS) as evaluation indicators. Specifically, *P* represents the ratio of the predicted algorithm area to the actual detection area and *R* represents the proportion of accurately predicted categories to the total required categories. mAP is used to evaluate the performance of multiple classifiers. FPS is used to evaluate the speed of object detection in terms of the number of images that can be processed per second. The volume of network parameters indicates that a smaller model size means lower memory usage. The core formula for evaluating indicators is as follows:

(9) $$Precision=\frac{TP}{TP+FP}$$

(10) $$Recall=\frac{TP}{TP+FN}$$

(11) $$AP=\int_{0}^{1}p\left(r\right)dr$$

(12) $$mAP=\frac{1}{N}\sum AP_{i}$$

where *N* represents the category overview, TP represents the number of correctly identified positive samples, FP represents the number of false-positive negative samples, FN represents the number of missed positive samples, and TN represents the number of correctly identified negative samples. AP and mAP represent single-class accuracy and average accuracy, respectively.

### 4.3. Ablation Experiment

The table uses a check mark symbol (√) to indicate the corresponding improvement strategies. Each set of experiments was conducted with the same training parameters.

Based on the comparative data in Table 3, it is verified that the use of the LE module improved the model’s mAP by 2.8%. Additionally, incorporating the SPD-Conv concept to reconstruct the backbone significantly increased the model’s FPS to 153.6 and the mAP value to 93.8%. The higher FPS enables the model to process images faster, achieving real-time detection and analysis, and reduces the latency between image capture and processing. The MLCA attention mechanism module, being lightweight, contributed the most to the mAP improvement, reaching 94.1%. It can be observed that the SPD-Conv reconstruction caused a slight decrease in mAP. This may be because traditional convolution methods gradually expand the receptive field by increasing the network depth, whereas SPD-Conv changes the spatial dimensions of the feature maps in a less natural manner, affecting global information integration. However, reconstructing the backbone with SPD-Conv greatly enhanced the model’s FPS. As the aircraft detection task involves numerous ducts and a high workload, with stringent real-time requirements, faster defect identification can improve work efficiency and reduce downtime for maintenance. Combining the LE module, SPD-Conv, and MLCA attention mechanism to reconstruct the YOLOv8 model achieved a balance between accuracy, speed, and algorithm robustness. The results show an mAP of 96.3 and an FPS of 138.7.

**Figure 6:**
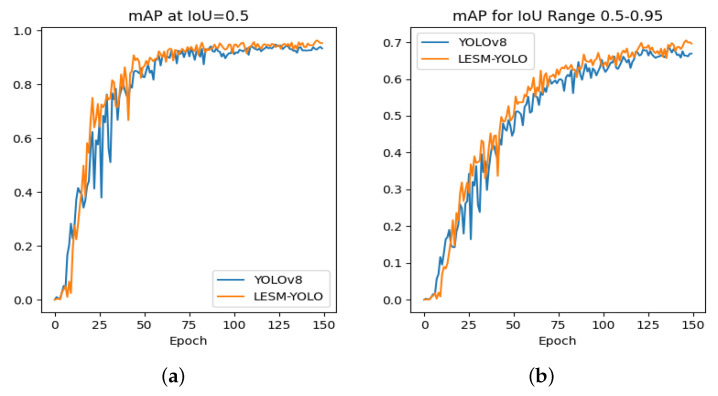
A comparison was conducted to evaluate the mAP values of the LESM-YOLO model against the original. (**a**) Comparison of mAP@0.5 and (**b**) comparison of mAP@0.5–0.95.

As shown in Figure 6, our model outperforms the original model in both mAP@0.5 and mAP@0.5:0.95 metrics. Initially, YOLOv8 shows a faster increase in mAP, but after 25 epochs, LESM-YOLO consistently achieves higher mAP@0.5 and mAP@0.5:0.95 metrics than the original model.

### 4.4. Interpretability Experiment

In deep learning, the confusion matrix is a tool used to evaluate the performance of classification models. It compares the model’s predictions with the true labels and categorizes them into four different scenarios: True Positive (TP), True Negative (TN), False Positive (FP), and False Negative (FN). Through the confusion matrix, the classification performance of the model for different classes can be obtained, providing us with a more comprehensive understanding of the model’s performance. In this experiment, we chose to compare the LESM-YOLO model with the YOLOv8 model and analyzed their confusion matrices to validate the performance of the proposed model.

**Figure 7:**
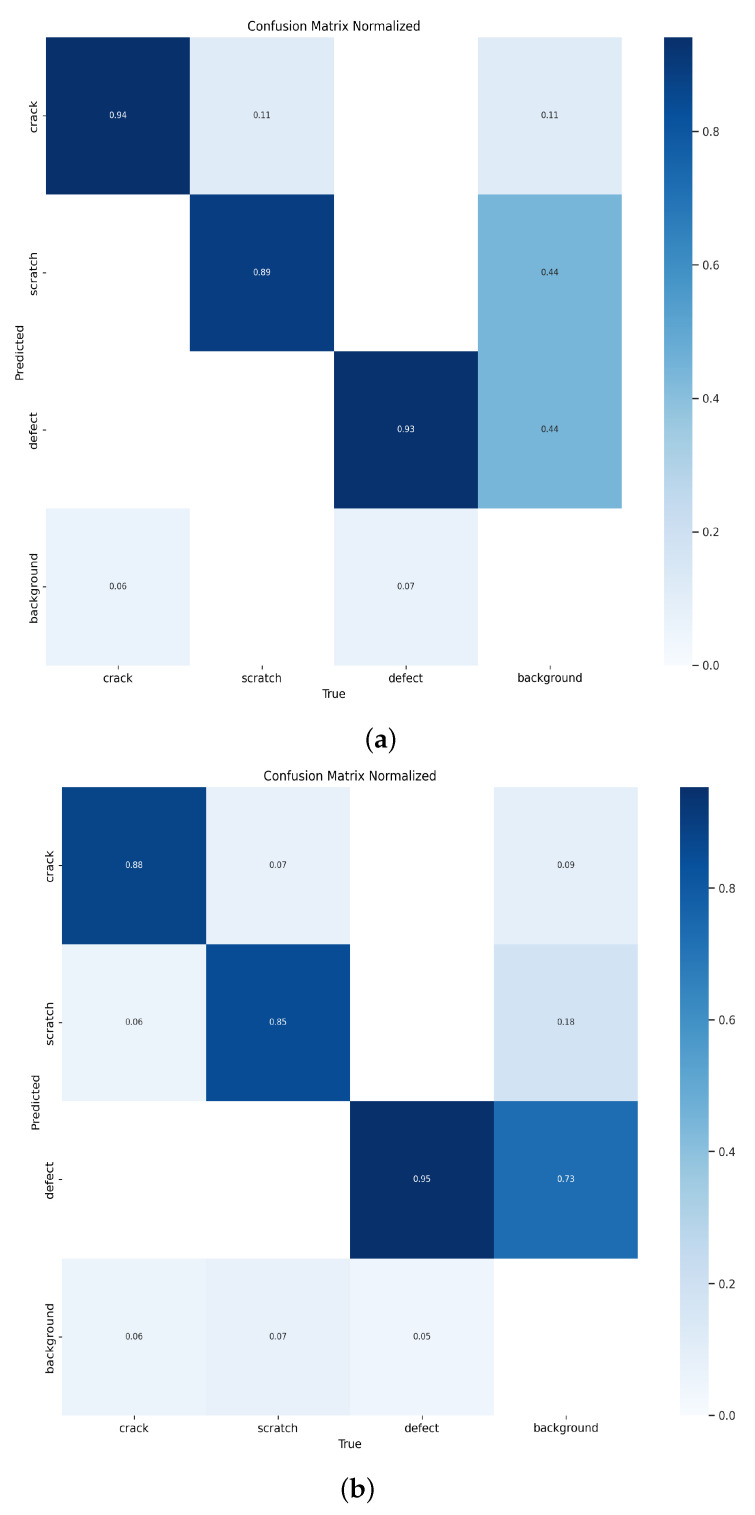
Confusion matrix of the LESM-YOLO model and YOLOv8 model. (**a**) Confusion matrix of LESM-YOLO. (**b**) Confusion matrix of YOLOv8.

From the Figure 7, it can be seen that when identifying scratch defects, the original YOLOv8 model had an accuracy of only 85%, while the LESM-YOLO model improved the accuracy by 4%. When identifying crack defects, the accuracy of the LESM-YOLO reached 94%, which is 6% higher than the original model. Therefore, the proposed LESM-YOLO significantly improves the accuracy of defect identification and exhibits a superior detection performance.

### 4.5. Comparison of Performance of Different Models

To evaluate the performance enhancement of the augmented model, this study conducted comparative experiments between the augmented model and various widely used object detection models. The selected models include two-stage anchor-based methods such as Faster R-CNN, as well as single-stage anchor-based methods such as SSD, YOLOv3, YOLOv4-tiny, YOLOv5, and YOLOv7, in addition to the Transformer-based YOLOS. Additionally, the experiments were conducted on the same dataset and under identical experimental conditions.

According to the results in Table 4, we found that, compared to the SSD model, our proposed LESM-YOLO model achieved a 4.67% improvement in the mAP performance metric and a 3.21 times speedup in detection. Additionally, compared to YOLOv8s and YOLOv7-tiny, with similar detection speeds, the model’s mAP increased by 5.4% and 8.87% respectively, achieving very satisfactory results. Compared to two-stage algorithms represented by Faster R-CNN, our LESM-YOLO model exhibits more significant advantages in both detection speed and accuracy.

Similarly, we conducted a performance comparison with the YOLOS (You Only Look at One Sequence) model [30]. YOLOS leverages the self-attention mechanism of the transformer to capture both global and local features in images. Our results demonstrate that our model outperforms YOLOS-Ti, achieving a 9% higher mAP50 score, which is a significant improvement. These findings indicate that the LESM-YOLO model exhibits superior performance and stability, with outstanding accuracy in detecting three types of defects.

**Figure 8:**
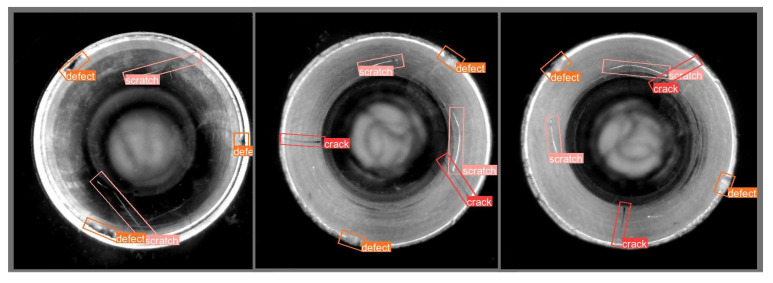
Actual detection results of the LESM-YOLO model.

Finally, our LESM-YOLO model was employed to detect the aircraft duct defect dataset. The detection results are shown in Figure 8. We utilized rotated boxes of different colors to represent the detection results for various types of defects. Specifically, red boxes indicate cracks, orange boxes indicate defects, and pink boxes indicate scratches.

## 5. Conclusions

Efficient and accurate detection is crucial for ensuring smooth aircraft operation. This paper proposes an aircraft duct defect detection model based on YOLOv8. In this approach, we incorporated a low-light enhancement module to effectively handle defect detection images captured under low-light conditions, thereby enhancing the accuracy and robustness of the model. Subsequently, we replaced the original convolutional layers with space-to-depth convolutions in the backbone section to reduce the parameter count of the model, making it suitable for deployment on edge detection devices while maintaining the defect detection accuracy. Finally, we improved the neck module of the original model by designing an MLCA-based neck, further enhancing the detection accuracy of the model. Through multiple experiments, we validated that our model can effectively complete aircraft duct defect detection tasks under low-light conditions with high quality.

## Figures and Tables

**Table 1:** Configuration and training environment.

Environmental Parameter	Value
System environment	Ubuntu 22.04
Deep learning framework	PyTorch 2.1.0
Cuda version	12.1
GPU	RTX 4090 (24 GB)
CPU	Intel(R) Xeon(R) Platinum 8352V CPU @ 2.10 GHz
Programming language	Python 3.10

**Table 2:** Hyperparametric configuration.

Hyperparameters	Value
Learning rate	0.01
Image size	640 × 640
Momentum	0.937
Batch size	4
Epoch	150
Weight decay	0.0005

**Table 3:** Ablation experiments with the modules.

LE-Module	SPD-Conv	MLCA	P	R	mAP	FPS
			87.5	85.7	89.9	140.3
√			89.6	91.3	92.7	135.8
	√		91.7	92.1	93.8	153.6
		√	91.4	89.9	94.1	128.9
√		√	95.5	90.1	97.1	124.4
√	√	√	94.8	92.8	96.3	138.7

^√^ indicates the corresponding improvement strategy.

**Table 4:** Comparison of detection performance among different models.

Models	Crack AP(%)	Scratch AP(%)	Defect AP(%)	mAP50	FPS
Faster-RCNN	80.89	72.49	73.81	75.73	9.6
SSD	94.34	89.86	90.69	91.63	43.2
YOLOv3	85.15	81.06	83.21	83.14	54.1
YOLOv4-tiny	81.81	77.54	80.02	79.79	145.3
YOLOv5	88.93	84.82	86.41	86.72	98.2
YOLOv7-tiny	90.13	84.63	87.53	87.43	102.3
YOLOS-Ti	88.64	85.18	86.67	86.83	113.6
YOLOv8s	90.82	86.76	95.12	90.90	140.3
Our Model	97.71	94.45	96.74	96.30	138.7

## Data Availability

The data that support the findings of this study are available from the corresponding author.
